# Diagnostic and prognostic value of CSF neurofilaments in a cohort of patients with motor neuron disease: A cross‐sectional study

**DOI:** 10.1111/jcmm.16240

**Published:** 2021-02-20

**Authors:** Delia Gagliardi, Irene Faravelli, Megi Meneri, Domenica Saccomanno, Alessandra Govoni, Francesca Magri, Giulia Ricci, Gabriele Siciliano, Giacomo Pietro Comi, Stefania Corti

**Affiliations:** ^1^ Dino Ferrari Centre Department of Pathophysiology and Transplantation (DEPT) University of Milan Milan Italy; ^2^ Fondazione IRCCS Ca' Granda Ospedale Maggiore Policlinico, Neurology Unit Milan Italy; ^3^ Neurological Clinics Department of Clinical and Experimental Medicine University of Pisa Pisa Italy; ^4^ Fondazione IRCCS Ca' Granda Ospedale Maggiore Policlinico, Neuromuscular and Rare Disease Unit Milan Italy

**Keywords:** amyotrophic lateral sclerosis, biomarkers, cerebrospinal fluid, motor neuron disease, neurofilaments, spinal muscular atrophy

## Abstract

Motor neuron disease (MND) is a rare group of disorders characterized by degeneration of motor neurons (MNs). The most common form of MND, amyotrophic lateral sclerosis (ALS), is an incurable disease with a variable rate of progression. The search of robust biomarkers able to discriminate among different ALS forms is paramount to properly stratify patients, and to identify those who could most likely benefit from experimental therapies. Phosphorylated‐neurofilament heavy chain (p‐NfH) and neurofilament light chain (NfL) are neuron‐specific components of the cytoskeleton and may represent reliable markers of neuronal injury in neurological disorders. In this study, we described our cohort of ALS patients in order to investigate whether and how cerebrospinal fluid (CSF) p‐NfH and NfL levels may reflect progression rate, MN involvement and the extent of neurodegeneration. CSF p‐NfH and NfL were significantly increased in ALS compared with healthy and disease controls, including patients with other forms of MND, and were higher in patients with more aggressive disease course, reflecting progression rate. We also evaluated neurofilament diagnostic accuracy in our centre, identifying with high sensitivity and 100% specificity cut‐off values of 0.652 ng/mL for CSF p‐NfH (*P* < .0001) and of 1261 pg/mL for NfL (*P* < .0001) in discriminating ALS from healthy controls. CSF neurofilaments were significantly correlated with ALS progression rate. Overall, CSF neurofilaments appear to reflect the burden of neurodegeneration in MND and represent reliable diagnostic and prognostic biomarkers in ALS.

## 1 INTRODUCTION

The term motor neuron disease (MND) defines a rare group of neurodegenerative disorders characterized by selective loss of motor neurons (MNs). According to the degree of MN involvement, the large spectrum of MND ranges from diseases presenting with pure upper motor neuron (UMN) signs, such as primary lateral sclerosis (PLS), and others affecting uniquely lower motor neurons (LMNs), including progressive muscular atrophy (PMA).

Amyotrophic lateral sclerosis (ALS), the most common form of MND, is a mainly sporadic adult‐onset neurodegenerative disorder, which affects both UMNs and LMNs. ALS patients suffer from muscle weakness and atrophy, variable severity of cognitive impairment, and usually die within 3‐5 years from symptom onset because of respiratory failure. However, disease duration is highly variable among patients, ranging from few months to more than 20 years.^1^ Spinal muscular atrophy (SMA) is an autosomal recessive neuromuscular disorder with the involvement of LMNs. Differently from ALS, clinical phenotype and disease duration of SMA are well‐characterized and largely rely on the number of copies of survival motor neuron 2 (*SMN2*) gene, which compensate for the lack of its paralogue *SMN1*.^2^ Specifically, although patients with SMA type 1 and type 2 do not reach several developmental motor milestones and have reduced survival, SMA type 3 patients have later disease onset and maintain the ability to ambulate at least until puberty.^3^

Significant clinical, genetic and pathological heterogeneity in MND has made early identification and prediction of disease course extremely complex. In this context, biomarkers are urgently needed not only for earlier diagnosis and prognosis estimation, but also for monitoring therapeutic trial efficacy.

Neurofilaments (Nfs) are neuron‐specific components of the cellular cytoskeleton, with a fundamental role in the stabilization and polarization of neurons.^4^ Following axonal injury, Nfs are released in the extracellular space and can be detected in the blood and cerebrospinal fluid (CSF). As Nfs levels have been shown to correlate with the severity of axonal damage,^5^ they could be a reliable marker of neuronal injury in neurological disorders. Because of its proximity to the central nervous system (CNS), the CSF contains greater Nf concentrations than serum, providing the most suitable source for Nf measurement and study.

In this work, we investigated whether CSF p‐NfH and NfL levels may reflect progression rate, MN involvement and burden of neurodegeneration in our cohort of ALS patients. Moreover, we evaluated the diagnostic accuracy of CSF p‐NfH and NfL levels in these patients, exploring their correlation with disease course and severity. Finally, we included our previously described cohort of SMA type 3 patients ^6^ in order to analyse the differences in Nfs levels between these two extremes of the MND spectrum.

## 2 MATERIALS AND METHODS

### 2.1 Patient evaluation

Patients were recruited at the Neurology Unit of Fondazione IRCCS Ca’ Granda Ospedale Maggiore Policlinico di Milano. The study was conducted in accordance with the Code of Ethics of the World Medical Association (Declaration of Helsinki) and its later amendments and with national legislation and institutional guidelines. The patients provided written informed consent for clinical data and biological sample collection. ALS diagnosis was formulated according to the El Escorial Revised and Awaji‐Shima diagnostic criteria,^7^, ^8^ and lumbar puncture (LP) for CSF collection was performed during the diagnostic assessment. Symptom onset was defined as first patient‐reported weakness, and disease duration was estimated at the time of presentation of medical attention. To uniform the heterogeneous disease duration, progression rate was calculated as 48 minus the ALS Functional Rating Scale–Revised (ALSFRS‐R) score at the time of presentation, divided by disease duration from symptom onset.^9^ ALS patients with progression rate less than 0.5, between 0.5 and 1.0, and more than 1.0 (points/month) were defined as slow‐, intermediate‐ and fast‐progressing ALS, respectively. CSF neurofilament levels were measured with commercially available enzyme‐linked immunosorbent assay (ELISA) kits (p‐NfH; Euroimmun, Lubeck, Germany, and NfL; UmanDiagnostics AB, Umea, Sweden) according to the manufacturer's instructions. Neurofilament levels were also assessed on CSF samples derived from patients ultimately diagnosed with functional or not neurological diseases. Moreover, patients with different neurological conditions and MND other than ALS were included. As previously reported,^6^ the values of Nfs below the lower limit of quantification were approximated to half of the concentration of the lowest calibrator (50 pg/mL and 0.0625 ng/mL for NfL and p‐NfH, respectively).

### 2.2 Statistical analysis

Baseline characteristics were analysed through descriptive statistics. Continuous variables were reported as mean ± standard deviation (SD), and categorical variables were represented as relative frequencies and percentages. Between‐group comparisons were performed with the Mann‐Whitney and Kruskal‐Wallis tests. Spearman's correlation coefficient was used to assess the association between variables. Binomial logistic regression was performed to evaluate the predictive potential of CSF Nfs. Receiver operating characteristic (ROC) curves were generated to assess the diagnostic value of p‐NfH and NfL in ALS patients compared with controls. Best cut‐off values were calculated with Youden's Index. Univariable binomial logistic regression was used to measure, in ALS patients, the association of fast progression compared with non‐fast progression (dependent variable) with either p‐NfH or NfL levels (independent variable); odds ratios (OR) and 95% confidence intervals (95% CI) were computed from regression coefficients and standard errors. Statistical analyses were performed with Prism (GraphPad) 8.3.1 Version.

## 3 RESULTS

### 3.1 Demographic and clinical features

We enrolled 32 patients with ALS with mean age at presentation of 65.7 (±10.8) years and mean disease duration at LP of 15.9 (±18.3) months. Demographic and clinical features of ALS patients are summarized in Table 1. The control group included 97 patients (51 males and 46 females), of which 18 were healthy controls (HC), 67 were diagnosed with other neurological disorders (disease controls, DC) and 12 patients were affected by SMA type 3. Age and sex of control population are listed in Table 2. Diagnoses in the DC group included central and peripheral nervous system inflammatory diseases (n = 15), peripheral neuropathies (n = 17), vascular disorders (n = 6) and neurodegenerative conditions other than MND (n = 29). Age of HC and DC was 42.2 (±20.7) and 63 (±14.9) years, respectively. Baseline features of patients with SMA type 3 were already described by Faravelli et al.^6^

**TABLE 1:** Demographic and clinical features of ALS patients

	Total (n = 32)	Male (n = 19)	Female (n = 13)
Age at presentation (years)	65.7 ± 10.8	66.3 ± 11.2	64.8 ± 10.6
Age at onset (years)	64.3 ± 10.5	65 ± 11.2	63.2 ± 9.6
Disease duration at LP (months)	15.9 ± 18.3	14.5 ± 10.3	18 ± 26.4
Site of onset, n (%)			
Spinal	25 (78.1)	16 (84.2)	9 (69.2)
Bulbar	7 (21.9)	3 (15.8)	4 (30.8)
Predominant signs, n (%)			
UMN	4 (12.5)	2 (10.5)	2 (15.4)
LMN	14 (43.7)	8 (42.1)	6 (46.1)
Both	14 (43.8)	9 (47.4)	5 (38.5)
Progression rate, n (%)			
Slow	15 (46.9)	11 (57.9)	4 (30.8)
Intermediate	10 (31.2)	3 (15.8)	7 (53.8)
Fast	7 (21.9)	5 (26.3)	2 (15.4)

Note

Mean ± SD and number (%), as appropriate. LMN: lower motor neuron; LP: lumbar puncture; UMN: upper motor neuron.

**TABLE 2:** Demographic features of controls

	Total (n = 97)	HC (n = 18)	DC (n = 67)	SMA type 3 (n = 12)
Age (years)	60 (35‐72)	38.5 (22.7‐54)	65 (55‐75)	28.5 (15‐34.8)
Sex, n (%)				
Male	51 (52.6)	7 (38.9)	36 (53.7)	8 (66.7)
Female	46 (47.4)	11 (61.1)	31 (46.3)	4 (33.3)

Note

Median and interquartile range [IQR], as appropriate. DC: disease controls; HC: healthy controls; SMA: spinal muscular atrophy.

### 3.2 CSF neurofilaments in MND versus controls

**FIGURE 1:**
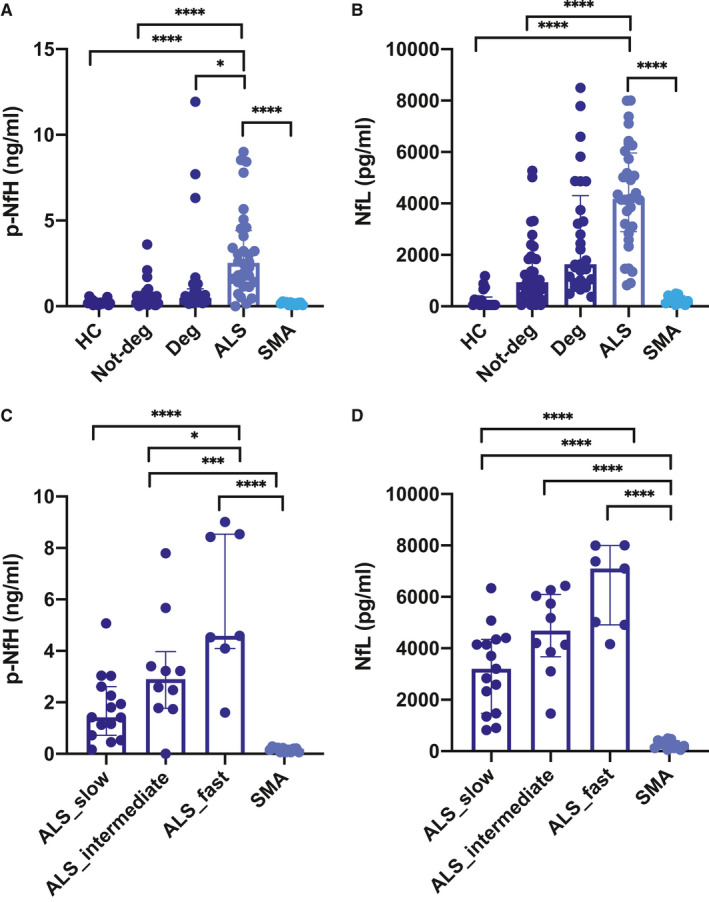
CSF neurofilament levels in ALS patients and controls. CSF p‐NfH and NfL levels according to disease type (A, B) and rate of progression (C, D). Individual data points are reported along with median [IQR]. Between‐group comparisons were performed with one‐way ANOVA or the Kruskal‐Wallis test, as appropriate. Follow‐up tests (Tukey's and Dunn's test) were carried out in case of significant differences

CSF p‐NfH and NfL levels were significantly increased in ALS patients (3.107 ng/mL and 4333.6 pg/mL, respectively) compared with HC (0.223 ng/mL, *P* < .0001, and 272.5 pg/mL, *P* < .0001), DC (0.917 ng/mL and 1908.2 pg/mL, *P* < .0001) and SMA type 3 patients (0.164 ng/mL, *P* < .0001, and 259.7 pg/mL, *P* < .0001) (Figure 1A, B). DC was further divided into patients with neurodegenerative disorders (Deg, n = 29) and without neurodegenerative disorders (Not‐deg, n = 38). Both CSF p‐NfH and NfL levels were significantly different in ALS compared with Not‐deg (0.538 ng/mL, *P* < .0001, and 1300.45 pg/mL, *P* < .0001, respectively), whereas only CSF p‐NfH levels were significantly increased in ALS versus Deg (1.413, *P* = .019).

Higher CSF p‐NfH and NfL levels were observed in ALS patients with fast progression rate compared to those with intermediate (*P* = .0149 for p‐NfH) and slow progression rate (*P* < .0001 for both p‐NfH and NfL) (Figure 1C, D). Moreover, both CSF p‐NfH and NfL levels were significantly lower in SMA type 3 patients compared with intermediate‐progressing (3.19 ng/mL, *P* = .0008 and 4641.29 pg/mL, *P* < .0001) and fast‐progressing ALS patients (5.827 ng/mL, *P* < .0001 and 6369.44 pg/mL, *P* < .0001).

### 3.3 CSF neurofilaments in ALS patients

**FIGURE 2:**
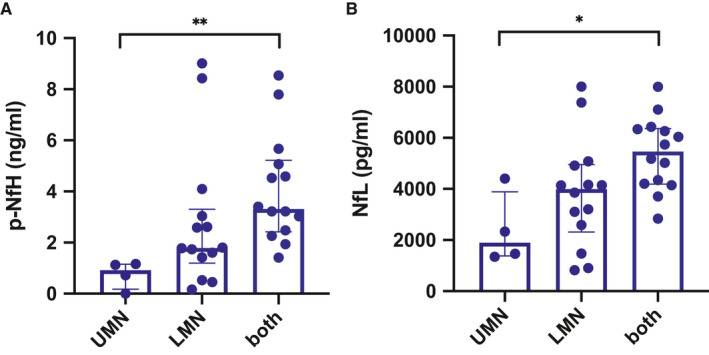
CSF neurofilament levels in ALS patients according to MN involvement. P‐NfH (A) and NfL (B) levels in patients with upper motor neuron (UMN) involvement, lower motor neuron involvement (LMN) or both. Between‐group comparisons were performed with one‐way ANOVA or the Kruskal‐Wallis test, as appropriate. Follow‐up tests (Tukey's and Dunn's test) were carried out in case of significant differences

To investigate the relationship between CSF Nf levels and the degree of MN involvement, ALS patients were classified on the basis of predominant upper motor neuron (UMN) and lower motor neuron (LMN) involvement, or both. We found a significant increase in CSF levels of both p‐NfH and NfL (*P* = .004 and *P* = .0156, respectively) in patients with MN involvement in comparison with those characterized by predominant UMN involvement (Figure 2A, B).

### 3.4 CSF neurofilaments are reliable diagnostic biomarkers in ALS

**FIGURE 3:**
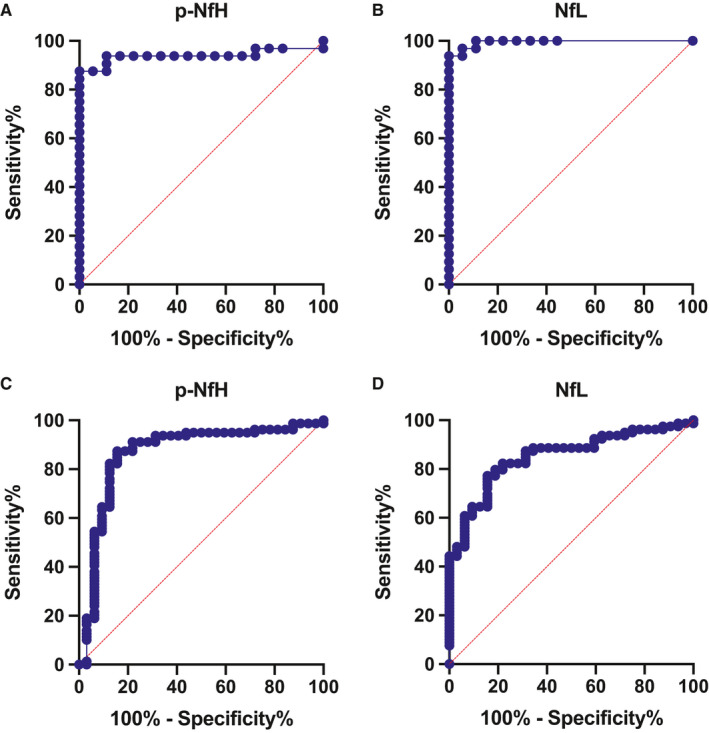
ROC analysis. Accuracy of CSF p‐NFH and NfL for the diagnosis of ALS vs HC (A, B); accuracy of CSF p‐NFH and NfL for the diagnosis of ALS vs DC + SMA type 3 (C, D)

We employed ROC analysis to assess to what extent CSF Nfs were able to discriminate between the ALS and control groups. Both CSF p‐NfH and NfL displayed a high accuracy for ALS diagnosis when compared to HC (AUC 0.939, *P* < .0001, and AUC 0.995, *P* < .0001, respectively) (Figure 3A, B), and the best cut‐off values were 0.652 ng/mL for CSF p‐NfH (sensitivity 87.5%, specificity 100%) and 1261 pg/mL for CSF NfL (sensitivity 93.75%, specificity 100%).

Comparing ALS with DC and SMA type 3 patients, CSF p‐NfH and NfL maintained a high diagnostic accuracy (AUC 0.867, *P* < .0001, and AUC 0.853, *P* < .0001, respectively) (Figure 3C, D). In this setting, a cut‐off value of 1.069 ng/mL for p‐NfH discriminated with 87.3% sensitivity and 84.4% specificity ALS from DC and SMA type 3 patients. Similarly, a cut‐off value of 1454 pg/mL for NfL discriminated with 64.6% sensitivity and 90.6% specificity ALS from the other diagnoses.

### 3.5 CSF neurofilaments are associated with progression rate in ALS patients

**FIGURE 4:**
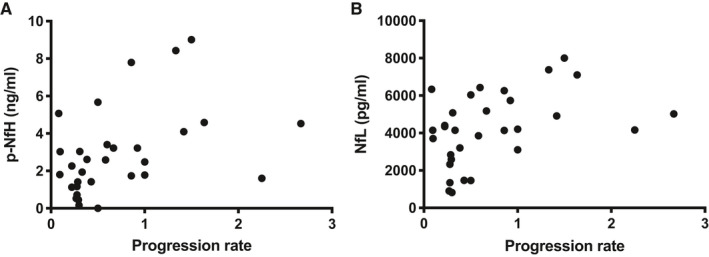
CSF neurofilaments correlate with ALS progression rate. A‐B, Both CSF p‐NfH and NfL significantly correlate with progression rate (*P* < .0097 and *P* < .0125)

We investigated whether CSF Nfs reflected the progression rate in ALS patients; we found a significant correlation between both p‐NfH (r = 0.443, *P* = .0125) and NfL levels (r = 0.4574, *P* = .0097) and progression rate in ALS cohort (Figure 4A, B).

Moreover, we performed logistic regression analyses using fast progression and non‐fast progression as a binary outcome. Both higher CSF p‐NfH and NfL levels were significantly associated with fast‐progressing ALS (OR 1.834, 95% CI 1.246‐3.203, *P* = .008, and OR 1.006, 95% CI 1.002‐1.015, *P* = .033, respectively).

## 4 DISCUSSION

In this work, we have reported the results of CSF Nf measurements in our cohort of ALS patients, highlighting their diagnostic and prognostic value. Limitations of the present study include the small number of patients and the heterogeneity of conditions among disease controls. However, we observed that ALS patients showed the highest value of Nf levels compared with healthy controls and other neurological conditions, including other MNDs such as SMA type 3.

Increasing evidence has suggested that p‐NfH and NfL hold diagnostic and prognostic potential in MNDs, particularly in ALS.^10^ Recent studies have demonstrated that CSF p‐NfH and NfL levels can discriminate ALS patients from HC and mimics.^11^, ^12^, ^13^, ^14^, ^15^ Specifically, CSF p‐NfH appears to be highly specific for MND and might be useful complementary tools in aiding early diagnosis. In addition, both CSF p‐NfH and NfL seem to correlate with the extent of clinical MN involvement ^14^, ^15^, ^16^ and their levels at baseline predict disease progression and survival,^12^, ^17^, ^18^ providing valuable insights into outcome prediction.

Findings from the present study have confirmed and expanded available literature data on utility of wet biomarkers in MND. Specifically, in our cohort, both p‐NfH and NfL presented elevate accuracy in discriminating ALS from HC, resulting in 100% specificity. However, when comparing ALS with DC and SMA type 3 patients, sensitivity and specificity were reduced, to a greater extent for NfL. This is likely because of the presence of neurodegenerative conditions among DC, and it is supported by findings on NfL levels that are not significantly different between ALS and degenerative group.

So far, a few studies have explored the role of CSF Nfs as clinical biomarkers in SMA.^15^, ^16^, ^17^, ^18^, ^19^ Increased concentration of NfL in serum and CSF and p‐NfH in plasma has been reported in SMA type 1 patients,^19^, ^20^, ^21^ likely reflecting a high burden of neurodegeneration. Conversely, patients with SMA type 3 did not show elevated CSF p‐NfH and NfL levels, which were comparable to the ones of healthy controls.^6^, ^19^, ^22^

To our knowledge, this is the first study, which intends to compare CSF Nf levels in two forms of MND affecting adult patients, ALS and SMA type 3. MN loss in ALS affects both upper and lower MNs and proceeds in a time window ranging from few months to decades from symptom onset. Starting in childhood, SMA type 3 is characterized by a lengthy disease course and a slow disease progression. Moreover, differently from ALS, it selectively affects LMNs in brainstem and spinal cord. Interestingly, p‐NfH levels were not significantly different between slowly progressing ALS and SMA type 3 patients. These data confirm the strict correlation between Nfs and neurodegeneration rate. Fast‐progressing ALS and intermediate‐progressing ALS are characterized by a tumultuous degeneration of MNs, whereas SMA type 3 presents a slower progression rate. In addition, ALS patients presenting both upper and lower MN signs displayed higher levels of Nfs, suggesting that a higher burden of degenerating MNs is reflected by these biomarkers. Consistently, lower levels of CSF Nfs detected in adult SMA can be explained not only by the lower rate of neurodegeneration but also by selective LMN involvement.

NfL levels remained significantly different between slowly progressing ALS and SMA type 3 patients; this result was in line with the lower specificity displayed by NfL.

Both CSF p‐NfH and NfL correlated with the extent of MN involvement and progression rate in ALS. Fast‐progressing ALS patients displayed significantly elevated concentrations of both p‐NfH and NfL at baseline with respect to intermediate‐progressing ones, which showed a similar trend in comparison with slow‐progressing patients.

Overall, this work corroborates the role of CSF Nfs as reliable and promising biomarkers in ALS, aiding clinical diagnosis and providing an accurate estimation of disease progression.

## CONFLICTS OF INTEREST

The authors declare no conflict of interest.

## AUTHOR CONTRIBUTIONS

**Delia Gagliardi:** Conceptualization (equal); Data curation (equal); Formal analysis (equal); Investigation (equal); Visualization (equal); Writing‐original draft (equal). **Irene Faravelli:** Conceptualization (equal); Data curation (equal); Formal analysis (equal); Investigation (equal); Visualization (equal); Writing‐original draft (equal). **Megi Meneri:** Conceptualization (equal); Data curation (equal); Investigation (equal). **Domenica Saccomanno:** Investigation (equal). **Alessandra Govoni:** Investigation (equal). **Francesca Magri:** Investigation (equal). **Giulia Ricci:** Investigation (equal). **Gabriele Siciliano:** Supervision (equal). **Giacomo Pietro Comi :** Supervision (equal); Writing‐review & editing (equal). **Stefania Corti:** Supervision (equal); Writing‐review & editing (equal).
